# Arsenite Methyltransferase Is an Important Mediator of Hematotoxicity Induced by Arsenic in Drinking Water

**DOI:** 10.3390/w15030448

**Published:** 2023-01-22

**Authors:** Sebastian Medina, Haikun Zhang, Laura V. Santos-Medina, Zachary A. Yee, Kaitlin J. Martin, Guanghua Wan, Alicia M. Bolt, Xixi Zhou, Miroslav Stýblo, Ke Jian Liu

**Affiliations:** 1 Department of Pharmaceutical Sciences, The University of New Mexico College of Pharmacy, Albuquerque, NM 87131, USA; 2 Department of Biology, New Mexico Highlands University, Las Vegas, NM 87701, USA; 3 Department of Nutrition, Gillings School of Global Public Health, University of North Carolina at Chapel Hill, Chapel Hill, NC 27599, USA; 4 Department of Pathology, Stony Brook University, Stony Brook, NY 11794, USA

**Keywords:** arsenic, arsenite (As^III^), anemia, red blood cells, biotransformation, arsenic (+III oxidation state) methyltransferase (As3MT)

## Abstract

Chronic arsenic exposures via the consumption of contaminated drinking water are clearly associated with many deleterious health outcomes, including anemia. Following exposure, trivalent inorganic arsenic (As^III^) is methylated through a series of arsenic (+III oxidation state) methyltransferase (As3MT)-dependent reactions, resulting in the production of several intermediates with greater toxicity than the parent inorganic arsenicals. The extent to which inorganic vs. methylated arsenicals contribute to As^III^-induced hematotoxicity remains unknown. In this study, the contribution of As3MT-dependent biotransformation to the development of anemia was evaluated in male *As3mt*-knockout (KO) and wild-type, C57BL/6J, mice following 60-day drinking water exposures to 1 mg/L (ppm) As^III^. The evaluation of hematological indicators of anemia revealed significant reductions in red blood cell counts, hemoglobin levels, and hematocrit in As^III^-exposed wild-type mice as compared to unexposed controls. No such changes in the blood of *As3mt*-KO mice were detected. Compared with unexposed controls, the percentages of mature RBCs in the bone marrow and spleen (measured by flow cytometry) were significantly reduced in the bone marrow of As^III^-exposed wild-type, but not *As3mt*-KO mice. This was accompanied by increased levels of mature RBCS in the spleen and elevated levels of circulating erythropoietin in the serum of As^III^-exposed wild-type, but not *As3mt*-KO mice. Taken together, the findings from the present study suggest that As3MT-dependent biotransformation has an essential role in mediating the hematotoxicity of As^III^ following drinking water exposures.

## 1. Introduction

Exposure to inorganic arsenic through the consumption of contaminated drinking water is a global public health issue [1]. It is estimated that greater than 200 million people worldwide are exposed to arsenic in their drinking water at levels known to be associated with many deleterious health outcomes [1–6]. Many regions impacted by arsenic contamination are also disproportionately affected by anemia [7–9]. Anemia is a widespread hematological disorder that adversely impacts the health of more than a billion people worldwide [10–12]. Anemia is identified based on alterations to several hematological parameters, including red blood cell counts (RBCs), hemoglobin levels, and RBC volumes (Kassebaum et al., 2014; WHO, 2015). Studies in human populations with chronic arsenic exposures report strong associations between arsenic and anemia [3,7–9,11,13,14]; however, the underlying mechanistic basis of these interactions remains to be fully understood.

In drinking water, arsenic occurs primarily as arsenate (As^V^) or arsenite (As^III^) [15,16]. As^III^ is generally regarded as the most toxic inorganic arsenical [17], and once ingested, is biotransformed in the liver, kidneys, lungs, and testes through a series of oxidative methylation and reduction reactions catalyzed by the arsenic (+3 oxidation state) methyltransferase (As3MT) enzyme [18,19]. During this multistep process, As^III^ is converted into monomethylarsonic acid (MMA^V^), monomethylarsonous acid (MMA^III^), dimethylarsinic acid (DMA^V^), and dimethylarsinous acid (DMA^III^) [18,19].

The biotransformation of arsenic is dependent on the activity of As3MT [20,21]. Arsenic biotransformation has an essential role in detoxification but also produces intermediates with greater in vitro toxicity than the parent inorganic arsenicals [19,22]. Variations in *As3mt* produced by single-nucleotide polymorphisms result in interindividual differences in arsenic biotransformation and are associated with differential sensitivities to arsenic toxicity [14, 23–25].

RBCs develop primarily in the bone marrow through the process of erythropoiesis [26,27]. Erythropoiesis is tightly regulated and orchestrated dominantly by the actions of the hormone erythropoietin (EPO) [26,27]. In response to a variety of intrinsic and extrinsic cues, erythromegakaryocytic progenitor cells in the bone marrow differentiate toward the RBC lineage [26,28,29]. The first stage of committed RBC differentiation is burst-forming unit-erythroid cells (BFU-E), which in response to EPO, further differentiate into colony-forming unit-erythroid cells (CFU-E), proerythroblasts, basophilic erythroblasts, polychromatophilic erythroblasts, and orthochromatophilic erythroblasts, ultimately producing mature RBCs that exit the bone marrow and enter systemic circulation [26,27].

Previous findings from our laboratory show that in vivo drinking water exposures of mice to As^III^ suppress the differentiation of erythroid progenitor cells in the bone marrow, resulting in the development of anemia [30,31]. We have also shown in vitro that the impairment of erythropoiesis is likely mediated by As^III^-induced disruption of GATA-1 and GATA-2 function [31,32]. However, the extent that As^III^ directly mediates the suppression of erythropoiesis in vivo remains unknown. In the present study, we evaluated the contribution of the As3MT-dependent biotransformation to the arsenic-induced hematotoxicity identified in our previous experiments [14, 30–34]. This study provides novel information regarding the relative contribution of inorganic As^III^ vs. methylated metabolites to the development of As^III^-induced anemia. This information is critical for understanding how polymorphisms in *As3mt* that reduce As^III^ methylation capacity may influence the risk of anemia via the impairment of RBC production in the bone marrow.

## 2. Materials and Methods

### 2.1. Reagents

Sodium meta-As^III^ (NaAsO_2_, ≥99% purity, CAS 7784-46-5, Cat. No. S7400), Iscove’s Modified Dulbecco’s Medium, and cell-culture-grade water were purchased from Sigma-Aldrich (St. Louis, MO, USA). Dulbecco’s phosphate-buffered saline without Ca^+2^ or Mg^+2^ (DPBS-) and fetal bovine serum were purchased from Atlanta Biologicals (Flowery Branch, GA, USA). L-Glutamine (200 mM) and penicillin/streptomycin (10,000 (mg/mL)/10,000 (U/mL)) were purchased from Life Technologies (Grand Island, NY, USA). Serum-free, M3436 MethocultTM methylcellulose medium containing erythropoietin for mouse erythroid progenitor cells (Cat. No. 03436) was purchased from STEMCELL Technologies (Cambridge, MA, USA). The EPO Quantikine ELISA kit for mice (Cat. No. MEP00B) was purchased from R&D Systems (Minneapolis, MN, USA). Acridine orange/propidium iodide (AO/PI) (Cat. No. CS2–0106) was purchased from Nexcelom Bioscience (Manchester, UK). Flow cytometry antibodies, rat anti-mouse PE CD71 (clone R17217; Cat. No. 113803), rat anti-mouse Alexa647 TER119 (clone TER-119; Cat. No. 116218), and the Zombie Aqua Fixable Viability Stain (Cat. No. 423102) were purchased from Biolegend (San Diego, CA, USA).

### 2.2. Mice

All experiments involving mice were performed at the University of New Mexico (UNM) Health Science Center (HSC) in accordance with the protocols approved by the UNM Office of Animal Care Compliance Committee (Albuquerque, NM, USA). Wild-type, C57BL/6J, male mice (7 weeks of age) were purchased from Jackson Laboratory (Bar Harbor, MA, USA). Male *As3mt*-knockout (KO) mice on a C57BL/6 background were generously provided by Dr. Miroslav Styblo from the University of North Carolina at Chapel Hill. *As3mt*-KO mice were originally developed by the Environmental Protection Agency, and their production is described in detail by Dobna et al., 2009 [35]. *As3mt*-KO mice were bred and maintained at the UNM HSC and utilized for experiments at 7 weeks of age. Male mice were exclusively utilized for this study to reduce the complications associated with gender differences and to facilitate comparisons with our previous findings from in vivo and in vitro studies using male mice [30–33].

### 2.3. Drinking Water Exposures

Wild-type and *As3mt*-KO mice were housed 2 or 3 per cage and exposed via their drinking water to the control (tap water from the UNM HSC in Albuquerque, NM, which was reported between 2015 and 2020 to contain approximately 6–8 μg/L (ppb) total arsenic; see https://ehs.unm.edu/environmental-affairs/drinking-water-quality.html) or 1 mg/L (ppm) As^III^ for 60 days (*n* = 5 mice/group). The duration of exposure was selected based on the circulating half-life of mature RBCs, which in mice is approximately 45 days; i.e., the total circulating RBC pool will be entirely replenished by newly produced cells after 45 days [26,27].

Stock solutions of As^III^ were prepared using cell-culture-grade water and diluted weekly into mouse drinking water pouches to yield the desired dosing concentration of 1 ppm As^III^. The arsenic concentrations in the stock solutions and dosing water (i.e., water collected from the mouse cages post-dosing) were verified by inductively coupled plasma-mass spectrometry [36]. Drinking water intake was monitored by weighing the water pouches at the beginning and end of each week, using the change in weight as an estimation of water consumption (1 mL *≈* 1 g H_2_O). The dose of As^III^ utilized in the present study was selected, taking into account that mice metabolize inorganic arsenic more efficiently than humans [37], resulting in the need to use higher doses in laboratory mouse studies. Although the dose of As^III^ used in this study exceeds the United States Environmental Protection Agency and World Health Organization drinking water standard of 10 ppb, it is well within the range of environmentally relevant arsenic levels experienced by many human populations around the world in their drinking water [3,5,7,9,11,38].

### 2.4. Collection of Blood, Preparation of Serum, and Hematology Analysis

The blood was collected and processed for hematology analysis or used for the preparation of the serum, as described by Medina et al., 2017 [30]. Whole blood was collected from each mouse at the time of euthanasia using cardiac puncture directly into either EDTA-coated 250 μL tubes (Cat. No. 2–03003, Fisher Scientific, Pittsburgh, PA, USA) or 1.5 mL microcentrifuge tubes for hematology analysis or serum preparation, respectively. An Abaxis VetScan HM5 hematology analyzer (Abaxis, Union City, CA, USA) was used for the analysis of the hematology parameters (i.e., complete blood count and differential). The serum was prepared by clotting the blood at room temperature for 2 h followed by centrifugation at 2000× *g* for 30 min. The serum was collected and stored at −80 °C prior to analysis.

### 2.5. Isolation of Primary Mouse Bone Marrow Cells

Bone marrow cells were isolated from the femur and tibia sets of each mouse, as described previously [39]. In brief, the cells were flushed from the femur and tibia bones and resuspended in Isocove’s Modified Dulbecco’s Medium (IMDM) supplemented with 2% heat-inactivated fetal bovine serum (HI FBS), 2 mM L-glutamine, 100 mg/mL streptomycin, and 100 units/mL penicillin. Viability and cell counts were measured using acridine orange/propidium iodide (AO/PI) and a Nexcelom Cellometer Auto 2000.

### 2.6. Isolation of Primary Mouse Spleen Cells

The spleens were collected and weighed prior to the isolation of the cells, as previously described [40]. Briefly, in a 65 mm dish containing cold RPMI 1640 with 10% HI FBS, 2 mM L-glutamine, and 100 mg/mL penicillin/streptomycin, single-cell suspensions were prepared through the homogenization of each spleen between the frosted ends of two glass microscope slides (Fisher Scientific, Pittsburgh, PA, USA). The cell counts and viabilities were assessed using AO/PI and a Nexcelom Cellometer 2000.

### 2.7. Bone Marrow BFU-E

BFU-E assays were set up in accordance with version 3.4.0 of STEMCELL Technologies Technical Manual for Mouse Colony-Forming Unit Assays using MethoCult^™^ and as described previously [30–32]. The bone marrow cells from the femurs and tibias were suspended at 0.5 × 10^6^ cells/mL, and 300 μL of this solution (1.5 × 10^5^ cells) was added to 3 mL MethoCult M3436 (STEMCELL Technologies). The samples were thoroughly mixed by vortexing, and 1.1 mL (5.5 × 10^4^ cells) of each suspension was plated in duplicate in specially coated 35 mm culture dishes designed for methylcellulose assays (STEMCELL Technologies). Duplicate sample dishes were placed with one uncovered 35 mm dish containing cell-culture-grade water into a closed 100 mm dish and incubated for 14 days in a humidified incubator at 37 °C with 5% CO_2_. Following incubation, BFU-E colonies containing ≥30 cells were identified based on morphological characteristics and counted using an inverted microscope. BFU-E data are reported as the number of colonies per total viable cells recovered from the femur and tibia sets of each mouse, as described previously by Ezeh et al., 2016 [39].

### 2.8. Flow Cytometry

Erythroid progenitor cell subsets in the bone marrow and spleen were evaluated using flow cytometry based on CD71 and TER119 surface marker expression [14,30–33]. The bone marrow and spleen cells (1 × 10^6^ cells) were stained with 0.5 μL of Zombie Aqua Fixable Viability Stain in 100 μL DPBS^−^ for 15 min at room temperature in the dark. The cells were then washed with flow wash/stain buffer (DPBS with 2% heat-inactivated FBS and 0.09% sodium azide) and stained with 0.5 μg of CD71-PE − and Ter119-Alexa647 monoclonal antibodies for 30 min at room temperature in the dark. The samples were resuspended in 0.5 mL flow wash/stain buffer and analyzed using an Invitrogen Attune NxT Flow Cytometer (Carlsbad, CA, USA). Flow cytometry data were analyzed using FlowJo version 10 (FlowJo LLC, Ashland, OR, USA).

### 2.9. Mouse EPO ELISA

The EPO levels were measured in the serum using the Mouse Quantikine ELISA Kit for EPO (R&D Systems), as described previously [30]. The serum from each mouse and EPO standards (0, 47, 94, 188, 375, 750, and 1500 pg/mL) were assayed in triplicate, according to the directions provided with the kit. Monoclonal antibody conjugated to horse radish peroxidase was utilized for the detection of EPO in the samples. Colorimetric reactions were stopped by adding 0.25 N hydrochloric acid to each well, and absorbance was measured immediately at 450 nm and 540 nm using a SpectraMax^®^ 340PC microplate reader (Molecular Devices, Sunnyvale, CA, USA). The data were adjusted for optical imperfections by subtracting measurements at 540 nm from those at 450 nm. The absolute EPO levels were extrapolated using a four-parameter logistic standard curve.

## 3. Arsenic Speciation by Hydride Generation-Cryotrapping-Inductively Coupled Plasma-Mass Spectrometry

Arsenic species were measured in the bone marrow cells, spleen cells, and the plasma using hydride generation-cryotrapping-inductively coupled plasma-mass spectrometry (HG-CT-ICP-MS) according to previously established methodologies [1–3]. The bone marrow and spleen cells (5 × 10^6^ total cells) were lysed using ice-cold deionized water. The plasma samples were simply diluted with deionized water. Each sample was treated with 2% cysteine prior to analysis to reduce all arsenic species to their trivalent oxidation state. Pentavalent arsenic standards (>98% purity) treated with cysteine were used to generate calibration curves [4] to quantify total inorganic arsenic (As^V^ and As^III^), total methyl arsenic (MMA^V^ and MMA^III^), and total dimethyl arsenic (DMA^V^ and DMA^III^) The HG-CT-ICP-MS level of detection for methylated arsenic species was 0.04 pg, and for inorganic arsenic species, it was 2.0 pg.

### Statistics

The data were analyzed with Excel 16.65 and GraphPad Prism 9.4.0. FlowJo version 10 (FlowJo LLC) was utilized to process all flow cytometry data. Five mice from each genotype (*n* = 5/group) were placed into control (tap water) or exposure groups and were utilized for statistical analyses. The differences between the control and As^III^ exposure groups for either wild-type or *As3mt*-KO mice were evaluated using a one-way ANOVA followed by Tukey’s post hoc test at a significance level of *p* < 0.05. The results from all assays include two-three technical replicates and are representative of two independent experiments, with comparable results attained.

## 4. Results

### 4.1. As^III^ Exposure Does Not Significantly Modify Mouse Body Weights, Drinking Water Consumption, Bone Marrow or Spleen Cell Recoveries, or Spleen Weights in Wild-Type and As3mt-KO Mice

The objective of this study was to evaluate whether As^III^ exposure alone, in the absence of As3MT-mediated biotransformation, results in the development of anemia through the impairment of RBC differentiation in the bone marrow. Male wild-type and *As3mt*-KO mice were exposed continuously to 1 ppm As^III^ via their drinking water for 60 days. Throughout the duration of the 60-day study, no signs of overt toxicity were observed for either genotype, including no significant changes to body weight, weekly drinking water consumption, and spleen weights (Table 1). In addition, no significant differences in the numbers of total viable cells recovered from the bone marrow or spleens of each mouse were detected for either wild-type or *As3mt*-KO mice following exposure to As^III^ for 60 days (Table 1).

### 4.2. Hematological Indicators of Anemia Are Reduced Following As^III^ Exposure in Wild-Type, but Not As3mt-KO Mice

Following the 60-day As^III^ exposure, blood was collected from each mouse using cardiac puncture and utilized for the analysis of hematological indicators of anemia [10,30,38,41], including RBC counts, hematocrit (Hct), hemoglobin (Hgb) levels, mean corpuscular hemoglobin (MCH) levels, and mean corpuscular volumes (MCV) were measured using an Abaxis VetScan hematology analyzer (Table 2). Wild-type mice exposed to 1 ppm As^III^ had significantly lower RBC counts (*p* < 0.05), Hct (*p* < 0.01), and Hgb levels (*p* < 0.05) compared to the wild-type controls, but no such differences were identified in the *As3mt*-KO mice (Table 2). No statistically significant differences in RBC counts, Hct, Hgb levels, or MCVs were detected in the *As3mt*-KO mice following As^III^ exposure for 60 days (Table 2). These findings suggest that arsenic biotransformation, catalyzed by the As3MT enzyme, has an important role in mediating the hematotoxicity of As^III^, as evidenced by the reduced hematological indicators of anemia in wild-type but not *As3mt*-KO mice.

### 4.3. Abundance and Distribution of Arsenic Species in the Bone Marrow, Spleen, and Plasma of As^III^ Exposed Wild-Type and As3mt-KO Mice

The abundance and distribution of arsenic and intracellular arsenic species were measured in the bone marrow, spleen, and plasma of the control and As^III^-exposed wild-type and *As3mt*-KO mice using HG-CT-ICP-MS (Table 3). As^III^ exposure substantially increased total intracellular arsenic and inorganic arsenicals (iAs) in the bone marrow, spleen, and plasma collected from the wild-type or *As3mt*-KO mice (Table 3). Relative to the untreated controls, statistically significant increases (*p* < 0.05 or lower) in the levels of methylated (MAs) and dimethylated arsenicals (DMAs) were detected in all three tissues (bone marrow, spleen, and plasma) from wild-type mice (Table 3). Arsenic measured in the tissues of the control mice is likely contributed via small amounts of arsenic present in chow and drinking water (~6–8 ppb). As expected, iAs were most abundant across all three tissues (bone marrow, spleen, and plasma) in As^III^-exposed *As3mt*-KO mice (Table 3). Intriguingly, methylated and dimethylated arsenicals (MAs and DMAs) were the major forms of arsenic detected in the bone marrow and plasma of wild-type mice, whereas iAs were the dominant forms detected in the spleen (Table 3).

### 4.4. Attenuation of BFU-E Colony Formation in Wild-Type, but Not As3mt-KO Mice Following 60-Day As^III^ Exposure

**Figure 1.**
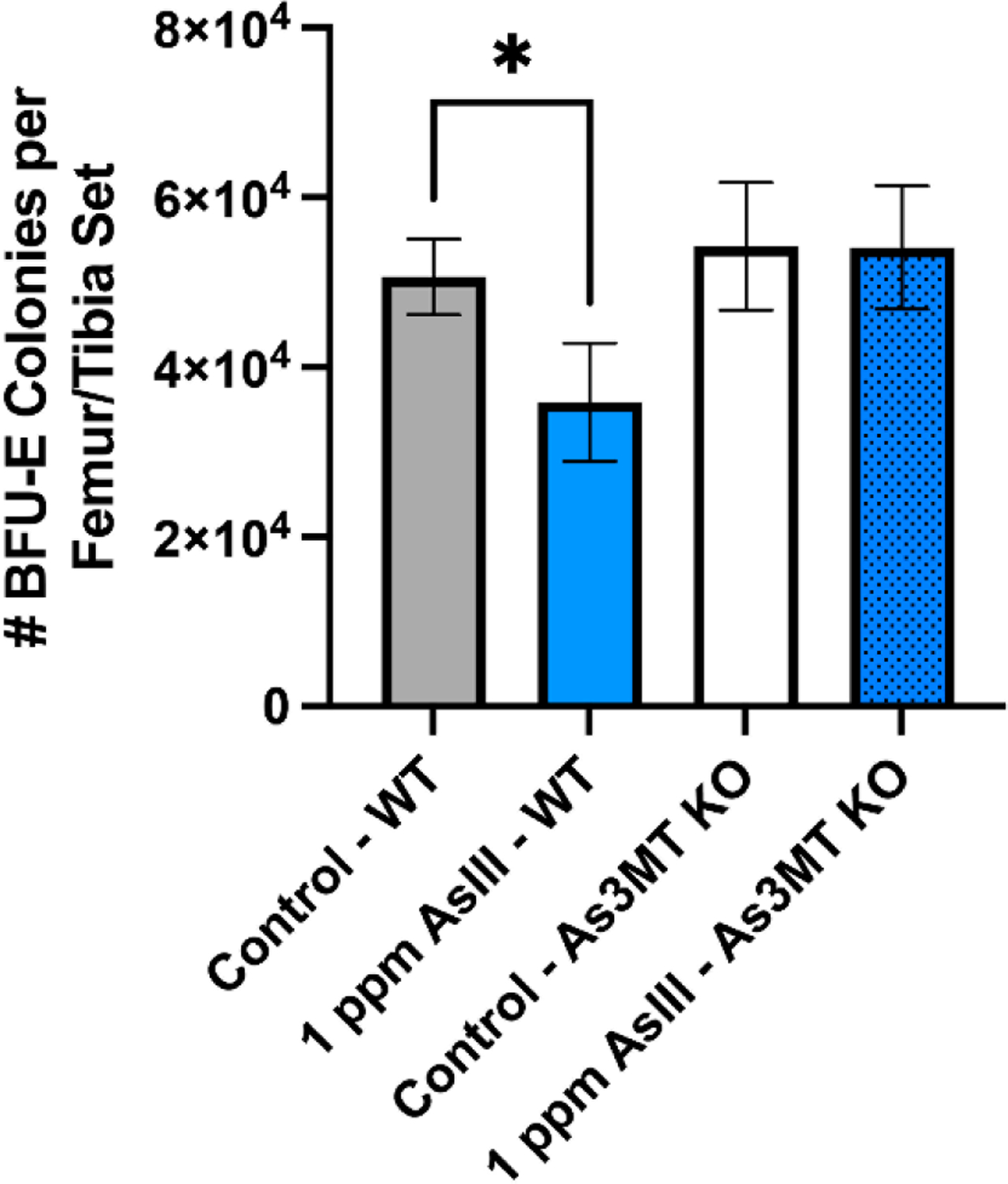
Drinking water exposure to As^III^ suppresses the formation of BFU-E colonies in wild-type, but not *As3mt*-KO mice following exposure to control (untreated, tap water) or 1 ppm As^III^ in their drinking water for 60 days. Number of BFU-E colonies (expressed relative to the number of viable cells recovered per femur and tibia set of each mouse) formed in EPO containing serum-free methylcellulose-based medium (i.e., Methocult M3436; STEMCELL Technologies) for 14 days. Data are expressed as mean ± SD of duplicate cultures per mouse. Statistically significant differences compared to untreated control in one-way ANOVA followed by Tukey’s post hoc test; *n* = 5 mice/group, *p < 0.05

To determine whether the anemia identified via hematology analysis was significantly contributed to by the loss of RBC progenitor differentiation, the colony-forming ability of BFU-E cells in the bone marrow was evaluated following the exposure of wild-type and *As3mt*-KO mice to 1 ppm As^III^ for 60 days (Figure 1). As^III^ exposure was found to significantly attenuate BFU-E colony formation in wild-type (*p* < 0.05) but not *As3mt*-KO mice (Figure 1). These findings suggest that As3MT-mediated biotransformation is important for the toxicity of As^III^ to early developing RBCs.

### 4.5. Early and Late-Stage Erythroblast Subsets Are Altered in the Bone Marrow of Wild-Type and As3mt-KO Mice Following Exposure to As^III^

**Figure 2.**
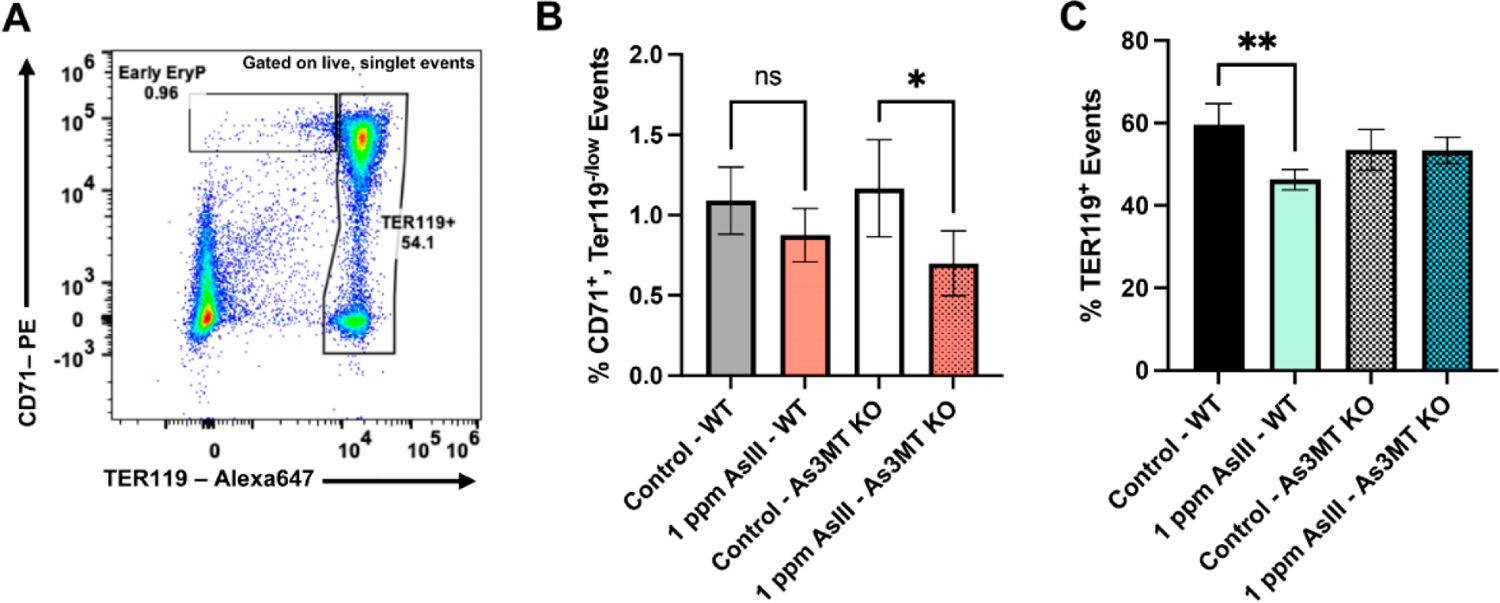
Percentages of early and late-stage erythroblasts were altered in the bone marrow of wild-type and *As3mt*-KO mice following 60-day drinking water exposure to 1 ppm As^III^. (**A**) Representative flow cytometry dot plot showing the gating strategy used to define early erythroid progenitors (Early EryP; CD71^+^, TER119^−/low^ events) or late-stage erythroblasts (CD71^+,−^, TER119^+^ events) in the bone marrow. Percentages of (**B**) early and (**C**) late-stage erythroblasts were measured by flow cytometry in bone marrow collected from wild-type and *As3mt*-KO mice following 60-day exposure to control (untreated, tap water) or or 1 ppm As^III^. Data are expressed as mean ± SD. Statistically significant difference compared to control in one-way ANOVA followed by Tukey’s post hoc test; *n* = 5 mice/group, *p < 0.05, **p < 0.01, non-statistically significant.

To further understand the ramifications of impaired arsenic biotransformation on the differentiation of RBCs, the proportion of early- and late-stage erythroid progenitor cells were evaluated in the bone marrow based on CD71 and TER119 surface marker expression using flow cytometry [14,30–33] (Figure 2A–C). The percentages of early-stage RBC progenitors showed a trend of reduction in wild-type mice and were significantly reduced in *As3mt-KO* mice (*p* < 0.05) following the 60-day As^III^ exposure (Figure 2B). As^III^ was also found to significantly decrease (*p* < 0.01) the percentage of late-stage, TER119^+^, RBC progenitor cells in the bone marrow of wild-type but not *As3mt*-KO mice (Figure 2C). This suggests that the suppressive effects of As^III^ on early RBCs are dependent on the As3MT-mediated biotransformation of As^III^.

### 4.6. As^III^ Exposure Increases Late-Stage, TER119^+^ Erythroblasts in the Spleens of Wild-Type, but Not As3mt-KO Mice

**Figure 3.**
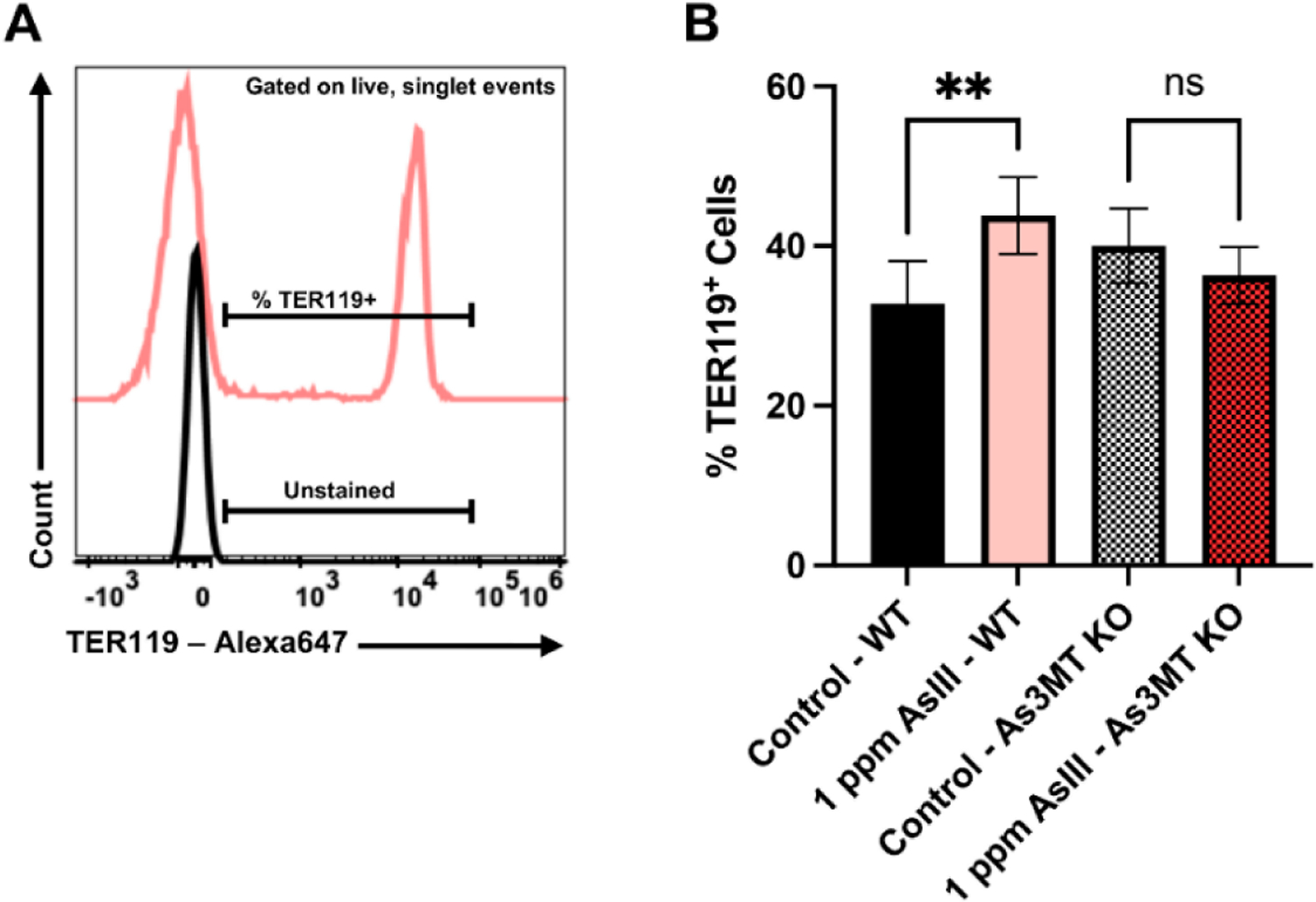
As^III^ exposure increases the percentage of mature TER119^+^ erythroblasts in the spleen of wild-type mice. (**A**) Representative flow cytometry histograms showing an unstained sample and the gating strategy used to enumerate the percentage of TER119^+^ erythroblasts and the spleen. (**B**) Percentages of TER119^+^ erythroblasts and the spleen wild-type and *As3mt*-KO mice following 60-day drinking water exposures control (untreated, tap water) or 1 ppm As^III^. Data are expressed as mean ± SD. Statistically significant difference compared to control in one-way ANOVA followed by Tukey’s post hoc test; *n* = 5 mice/group, **p < 0.01, ns = non-statistically significant.

The proportions of late-stage RBC progenitor cells in the spleens of wild-type and *As3mt*-KO mice were evaluated following exposure to As^III^ for 60 days based on TER119 surface marker expression using flow cytometry [14,30–33]. There was a significant increase (*p* < 0.01) in the percentage of late-stage TER119^+^ RBCs in the spleen of wild-type mice (Figure 3A,B). No significant alterations in the proportion of TER119^+^ RBCs were found in the spleens of *As3mt*-KO mice (Figure 3A,B). Taken together, these results suggest that suppressed RBC output from the bone marrow of wild-type mice induces erythropoietic stress, causing a compensatory increase in RBC production from the spleen. This further underscores the role of biotransformation, mediated by the As3MT enzyme, to the impaired differentiation of RBCs.

### 4.7. Levels of Circulating EPO Are Increased Following As^III^ Exposure in Wild-Type, but Not As3mt-KO Mice

**Figure 4.**
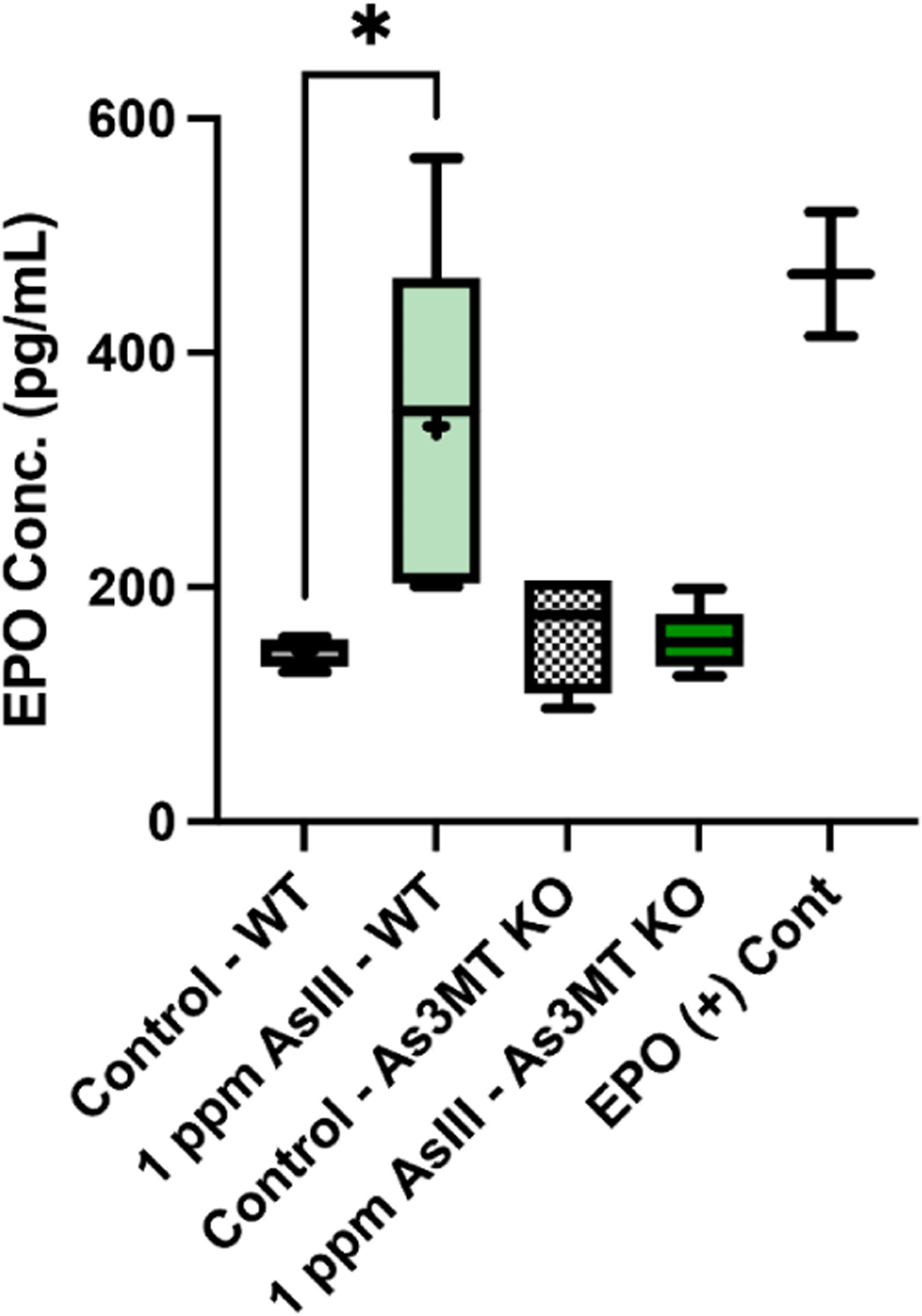
Elevated serum EPO levels in wild-type mice following drinking water exposure to As^III^. EPO concentrations were measured using the Mouse EPO Quantikine ELISA Kit (R&D Systems) in collected from wild-type and *As3mt*-KO mice following exposure to control (untreated, tap water) or 1 ppm As^III^ for 60 days.EPO positive (+) control was a standard prepared at a known concentration of 500 pg/mL. In the box plot, crosses indicate the mean and black horizontal lines indicate the median. Data are expressed as mean ± SD. Statistically significant difference compared to control in one-way ANOVA followed by Tukey’s post hoc test; *n* = 5 mice/group, *p < 0.05.

During instances of hypoxic stress, such as those produced by decreased RBC production from the bone marrow, the release of EPO from the kidney is increased. Increased EPO production promotes the survival and rapid expansion of the early erythroid progenitor pool in the bone marrow [26]. To further determine if the reduced hematological indicators of anemia and compromised production of mature RBCs from the bone marrow were accompanied by an increase in serum EPO levels, the concentration of EPO in the serum of wild-type and *As3mt*-KO mice was measured using the Mouse EPO ELISA Kit (R&D Systems). A significant increase (*p* < 0.05) in circulating EPO concentrations in the serum of 1 ppm As^III^-exposed wild-type mice was detected (Figure 4). Circulating EPO levels were not significantly altered in *As3mt*-KO mice following As^III^ exposure (Figure 4). Collectively, these findings suggest that the biotransformation of As^III^ produces erythropoietic stress, as indicated by suppressed RBC production in the bone marrow, increased production of mature RBCs from the spleen, and elevated levels of circulating EPO in wild-type but not *As3m*-KO mice.

## 5. Discussion

Millions of people worldwide are exposed to arsenic in their drinking water at levels known to produce numerous detrimental health outcomes, including cancer, cardiovascular disease, immune suppression, and anemia [1–3,5,14–16]. Despite our growing understanding of the mechanistic basis of arsenic-induced diseases, a significant knowledge gap remains in our understanding of the relative contribution of inorganic vs. organic arsenical species to the development of such ailments. To our knowledge, this is the first study to evaluate the effects of arsenic biotransformation on RBC progenitor cell development and anemia. Our findings provide novel evidence demonstrating a role for biotransformation in arsenic-induced anemia. We found that As^III^ exposure resulted in the development of anemia in an As3MT-dependent manner. In addition, the suppressive effects of As^III^ on the differentiation of RBC progenitors in the bone marrow were also found to be associated with the *As3mt* genotype.

The findings from our group have established that exposure to environmentally relevant levels of As^III^ results in the development of anemia via the suppression of erythropoiesis in the bone marrow [14,30–34]. In the present study, we found that the *As3mt* genotype has a critical role in mediating the hematotoxicity of arsenic. Intriguingly, the absence of the As3MT enzyme substantially reduced the effects of As^III^ on RBCs in the bone marrow, suggesting the process of biotransformation of inorganic arsenic (i.e., the production of methylated arsenical intermediates) is an important driver of hematotoxicity in vivo. Our lab and others report that MMA^III^ is more toxic than As^III^ in vitro [17, 42–44] and produces stronger suppressive effects to early developing RBCs in vitro [32]. This is significant because, in the present study, we found the predominant arsenic species in the bone marrow of As^III^-exposed wild-type mice were methylated arsenicals. This is consistent with our previous results showing that MMA^III^ is the major arsenical present in the bone marrow of mice exposed to As^III^ in their drinking water [40]. Dosimetry studies in *As3mt*-KO mice following drinking water exposure to As^III^ report similar arsenic metabolite profiles as those observed in this study, with the predominant arsenicals present in *As3mt*-KO mice being inorganic [45].

These findings suggest that arsenic metabolism may play a significant role in impairing erythropoiesis through the generation of MMA^III^, which in the absence of the As3MT enzyme, largely does not occur [45]. Numerous epidemiological studies in populations chronically exposed to arsenic have shown that arsenic methylation capacity is associated with elevated disease risk [46–52]. The proportions of excreted arsenic metabolites are indicative of arsenic methylation efficiency [24,37,53]. Higher levels of DMA relative to MMA in urine are indicative of more efficient arsenic metabolism and are associated with decreased toxicity [19,54]. MMA^III^ exposure may therefore be a significant contributing factor to anemia in people chronically exposed to arsenic. Consistent with this hypothesis, our previous studies revealed an inverse association between urinary MMA and RBC counts in a cohort of men from rural Bangladesh [38]. Additionally, findings from the present study identified significant impairments to erythropoiesis that were associated with the *As3mt*-genotype.

Our previous results show that As^III^ exposure results in the development of anemia via the suppression of RBC differentiation in the bone marrow of male C57BL/6J mice [30]. Consistent with these findings, the formation of BFU-E colonies and percentages of late-stage erythroblasts were reduced in wild-type mice. Whereas our previous studies focused on the role of arsenic exposure in the suppression of erythropoiesis and the development of anemia, the present study extends beyond these works by evaluating the role of As3MT-mediated biotransformation in the hematotoxicity of As^III^. Although BFU-E colony formation was not significantly altered in *As3mt*-KO mice, the percentages of early erythroid progenitor cells were significantly reduced. These findings suggest that different stages of early RBC differentiation may exhibit varying sensitivities to different arsenical species. The extent to which specific inorganic or organic arsenicals contribute to this phenomenon in vivo remains unclear but will be the focus of our ongoing investigations. The current study only indicates the critical role of arsenic methylation in As^III^-induced anemia. Whether methylation plays a similar role in other arsenic-mediated adverse health effects remains unknown and warrants further study.

Anemic conditions, such as those caused by impairment of bone marrow erythropoiesis, induce a physiological response mechanism known as stress erythropoiesis to cope with the loss of RBCs [55,56]. An important distinction between mice and humans is that the primary site of stress erythropoiesis in mice is the spleen, whereas, in humans, it occurs predominately in the bone marrow [55–57]. Consistent with the hypothesis, the wild-type mice in the present study demonstrated distinguishing factors of stress erythropoiesis, including elevated percentages of mature erythroblasts in the spleen and increased circulating EPO levels. These findings suggest that the As^III^-induced suppression of bone marrow erythropoiesis resulted in the activation of stress erythropoiesis in the spleen.

Taken together, the findings from the present study provide novel support for the role of biotransformation in arsenic-induced hematotoxicity and anemia. These results emphasize the importance of considering arsenic biotransformation as a mediator of toxicity in humans. This information is essential for understanding the molecular mechanisms by which arsenic exposure produces adverse health effects and provides a critical framework for understanding how biotransformation influences the risk of arsenic-associated anemias, which is critical for the development of potential preventative and therapeutic strategies in the future.

## Figures and Tables

**Table 1.** As^III^ exposure does not significantly alter body weight, weekly drinking water consumption, spleen weights, or bone marrow and spleen cell recoveries in male wild-type or *As3mt*-KO mice exposed via drinking water to control (tap water) or 1 ppm As^III^ for 60 days^a^.

	Wild-Type		*As3mt*-KO	
Parameter	Control	1 ppm As^III^	Control	1 ppm As^III^
Body Wt. (g)	32.1 ± 3.18	31.38 ± 1.12	29.50 ± 1.78	29.99 ± 3.23
Water Consumption (mL/mouse/wk)	21.00 ± 1.50	21.00 ± 4.00	17.00 ± 0.98	19.00 ± 2.00
Bone Marrow Cell Recovery				
(Total viable cells per	57.90 ± 6.38	51.14 ± 11.75	66.92 ± 8.24	63.26 ± 11.06
femur/tibia set, ×10^6^)				
Spleen Wt. (g)	80.80 ± 8.20	99.80 ± 18.49	79.4 ± 4.89	76.4 ± 7.30
Spleen Cell Recovery (Total viable cells, ×10^6^)	153 ± 15.3	150 ± 15.3	98.66 ± 15.30	109.24 ± 18.97

a Data are expressed as mean ± SD, *n* = 5.

**Table 2.** Alterations to hematological indicators of anemia (i.e., RBC counts, Hct, Hgb levels, MCH levels, and MCVs) measured in whole blood collected from male wild-type and *As3mt*-KO mice following drinking water exposure to control (tap water) or 1 ppm As^III^ for 60 days^a^.

	Wild-Type		*As3mt*-KO	
Parameter	Control	1 ppm As^III^	Control	1 ppm As^III^
RBC Count (×10^12^/L)	12.17 ± 0.13	11.21 ± 0.22***	11.66 ± 0.25	11.47 ± 0.75
Hct (%)	52.08 ± 0.51	49.1 ± 0.38***	48.56 ± 1.26	48.12 ± 3.47
Hgb (g/dL)	16.7 ± 0.47	16.12 ± 0.30*	14.96 ± 2.21	15.66 ± 1.21
MCH (pg)	13.98 ± 0.53	14.38 ± 0.46	13.58 ± 0.29	13.66 ± 0.30
MCV (fL)	43 ± 0.71	43.8 ± 0.84	41.8 ± 0.45	41.8 ± 0.45

a Data are expressed as mean ± SD, *n* = 5,

* *p* < 0.05,

*** *p* < 0.0001, in one-way ANOVA followed by Tukey’s multiple-comparison post hoc test.

**Table 3.** Arsenic species measured using HG-CT-ICP-MS in bone marrow cells, spleen cells, and plasma collected from male wild-type and As3mt-KO mice following exposure to control (tap water) or 1 ppm As^III^ for 60 days^a,b^.

Tissues	Genotype	Exposure		As Amount by Species (pg)		
			iAs	MAs	DMAs	Total As
Bone Marrow	Wild-type	Control	1.50 ± 0.82	0.22 ± 0.09	0.75 ± 0.15	2.46 ± 0.89
		1 ppm AsIII	4.80 ± 2.43	2.66 ± 2.14 *	23.23 ± 9.71 ****	30.69 ± 13.78
	*As3mt*-KO	Control	11.63 ± 1.02	0.37 ± 0.12	0.25 ± 0.12	12.25 ± 1.06
		1 ppm AsIII	134.27 ± 28.71 ****	1.55 ± 0.24	0.23 ± 0.09	136..05 ± 28.50****
Spleen	Wild-type	Control	6.68 **±** 4.82	0.19 **±** 0.13	0.26 **±** 0.14	7.14 **±** 4.97
		1 ppm AsIII	10.04 **±** 3.32	0.75 **±** 0.38 **	5.22 ± 1.61 ****	16.00 **±** 2.65
	*As3mt*-KO	Control	30.14 **±** 11.02	0.33 **±** 0.13	0.21 **±** 0.08	30.68 **±** 11.18
		1 ppm AsIII	89.33 **±** 17.79 ****	0.98 **±** 0.20**	0.14 **±** 0.05	90.45 **±** 17.90****
Plasma	Wild-type	Control	12.855 **±** 6.47	2.29 **±** 0.91	218.98 **±** 54.90	234.11 **±** 61.23
		1 ppm AsIII	92.32 **±** 39.29	117.6 **±** 3.56 ****	6956.75 ± 1495.13 ****	7166.57 **±** 1533.08****
	*As3mt*-KO	Control	243.74 **±** 142.48	2.44 **±** 1.32	1.49 ± 0.62	247.67 **±** 144.19
		1 ppm AsIII	1537.93 **±** 257.47****	26.33 **±** 2.14 ****	8.81 **±** 4.67	1573.77 **±** 254.08

a Data are expressed as mean ± SD, *n* = 5,

* *p* < 0.05,

** *p* < 0.01,

**** *p* < 0.0001, in one-way ANOVA followed by Tukey’s multiple-comparison post hoc test.

b Inorganic arsenicals (iAs; As^V^ and As^III^); methylated As (MAs; MMA^V^ and MMA^III^); demethylated As (DMAs; DMA^V^ and DMA^III^); Total As (iAs + MAs + DMAs).

## Data Availability

Data generated during the present study are available from the corresponding author (Sebastian Medina; semedina@nmhu.edu) upon reasonable request.
